# The homozygous long variant of *5-HTT* gene in congenital central hypoventilation syndrome is associated with a modified resting CO_2_ set point

**DOI:** 10.1186/s13023-026-04544-7

**Published:** 2026-08-12

**Authors:** Nathalie Couque, Benjamin Dudoignon, Plamen Bokov, Christophe Delclaux

**Affiliations:** 1 AP-HP, Hôpital Robert Debré, Département de génétique, INSERM NeuroDiderot, Paris, F-75019 France; 2 Université de Paris, AP-HP, Hôpital Robert Debré, Service de Physiologie Pédiatrique - Somnologie - CRMR Maladies respiratoires rares, INSERM NeuroDiderot, Paris, F-75019 France; 3 Service de Physiologie – Explorations Fonctionnelles - Somnologie Hôpital universitaire pédiatrique Robert Debré, 48, boulevard Sérurier, Paris, 75019 France

**Keywords:** Congenital central hypoventilation syndrome, Serotonin, *5-HTT* transporter polymorphism, CO_2_ chemosensitivity, CO_2_ set point

## Abstract

**Background:**

Central respiratory chemoreceptors are not definitively identified, but the retrotrapezoid nucleus is the main center whose absence is characteristic of congenital central hypoventilation syndrome (CCHS). Intense chemosensory stimuli activate circuits that are wake- or attention-promoting, such as the raphe, which modulates chemoreflexes. The main objective of our study was to assess whether residual central CO_2_ chemosensitivity in CCHS is related to serotonin transporter gene polymorphisms (homozygous long (ll) variant versus variants with short form, ss or sl). Twenty-three children with CCHS and PHOX2B pathogenic variants had peripheral (controller gain measurement) and central CO2 chemosensitivity assessment (hyperoxic, hypercapnic test), resting end-tidal PCO2 (PETCO2) measurement and 5-HTT polymorphism determination.

**Results:**

The percentages of the ll, ls, and ss genotypes were 5/23 (22%), 14/23 (61%) and 4/23 (17%), respectively, and were not influenced by *Phox2b* mutations. Both central (0.16 L/min/mmHg [0.02; 0.33] versus 0.21 [0.06; 0.41], *P* = 0.551) and peripheral (0.47 L/min/mmHg [0.026; 0.71] versus 0.67 [0.24; 1.26], *P* = 0.602) CO_2_ chemosensitivities were not significantly different when comparing the L group (*n* = 5) and the S group (*n* = 18), while their resting PETCO_2_ values were different; the L group presented a higher PETCO_2_ value than the S group did (49.3 mmHg [46.9; 53.0] versus 42.0 [36.7; 44.8], *P* = 0.014).

**Conclusions:**

The CO_2_ chemosensitivity of children with CCHS was not related to *5-HTT* transporter polymorphisms, whereas the ll homozygous variant contributed to resting alveolar hypoventilation.

## Background

Congenital central hypoventilation syndrome (CCHS) is a genetic neurodevelopmental disorder with alveolar hypoventilation as a hallmark symptom [1]. Whereas this hypoventilation is most prominent during sleep, spontaneous waking ventilation is common and can result from chemosensory stimuli that activate circuits that are wake- or attention-promoting, such as the raphe, which modulates the chemoreflexes [2].

Mice bearing a mutation in the paired-like homeobox 2B gene (*PHOX2B*), which causes CCHS in humans to die soon after birth from central apnea, while specifically lacking *PHOX2B*-expressing glutamatergic neurons located in the retrotrapezoid nucleus [3]. Casciato and colleagues recently observed in a preclinical model of CCHS, the retrotrapezoid nucleus conditional *PHOX2B* mutant mouse, that etonogestrel restored chemosensitivity in *PHOX2B* mutants in a nonsystematic way [4]. Histological differences between *PHOX2B* mutants with restored chemosensitivity and *PHOX2B* mutants without restored chemosensitivity indicated greater activation of serotonin neurons in the R. obscurus nucleus but no effect on residual *PHOX2B* cells in the retrotrapezoid nucleus. Finally, the increase in serotonergic signaling caused by fluoxetine application modulated the respiratory effect of etonogestrel differently between *PHOX2B* mutant mice and their wild-type littermates or their wild-type littermates OF1, a result that parallels the differences in the functional state of serotonergic metabolic pathways between these different mice [4]. This highlights the importance of the functional status of serotonin (5-HT) systems for an etonogestrel-induced respiratory effect to occur in a CCHS context. This could explain why some patients with CCHS are sensitive to etonogestrel in terms of the ventilatory response to CO_2_, whereas others are not [5]. This could also explain why there is varying residual central CO_2_ chemosensitivity in patients with CCHS [6]. This hypothesis is all the worth testing given that known polymorphisms related to serotonergic neurotransmission [7] are involved in the clinical expression of diseases [8] and reactions to certain treatments [9]. Human 5-HT transporter (*5-HTT*) gene transcription is modulated by a common polymorphism in its upstream regulatory region. The short variant of the polymorphism reduces the transcriptional efficiency of the *5-HTT* gene promoter, resulting in decreased *5-HTT* expression and 5-HT uptake in lymphoblasts [10], whereas the long (l) allele drives a 2- to 3-fold higher rate of *5-HTT* gene transcription than the short (s) allele does. The transporter protein, by recycling serotonin, regulates its concentration in synapses and thus its effects. That is, the s allele corresponds to low serotonin uptake activity. The distributions of *5-HTT* genotypes in controls in the population from France revealed that the percentages of the ll, ls, and ss genotypes were 27% (95% confidence interval [CI]: 15–42), 50% (95% CI: 35–65) and 23% (95% CI: 12–37), respectively [8].

We recently confirmed the presence of residual central CO_2_ chemosensitivity in young subjects with CCHS, and the median [interquartile] value of the CO_2_ response slope was 0.27 [0.15; 0.36] L/min/mmHg [11]. Consequently, 25% of our subjects had an almost null response (slope < 0.15 L/min/mmHg), 50% had a decreased response (slope 0.15 to 0.36 L/min/mmHg), and 25% had a relatively preserved response (slope > 0.36 L/min/mmHg), which could be related to the differential expression of 5-HTT polymorphisms.

The main objective of our study was to assess whether residual central CO_2_ chemosensitivity is related to *5-HTT* gene polymorphisms, and the secondary objectives were to assess whether these polymorphisms influence the peripheral CO_2_ response and PCO_2_, which are other parameters of ventilatory control.

## Methods

The sample was composed of 23 patients with CCHS followed-up at our national reference pediatric center for rare diseases. All the patients had a confirmed pathogenic variant of *PHOX2B* with either a polyalanine repeat mutation (PARM) or non-PARM. Ethical approval was obtained from our Ethical Committee (Comité d’Evaluation de l’Ethique des Projets de Recherche de Robert Debré; No. 2023 − 694) in accordance with the Declaration of Helsinki. The parents and children were asked to provide informed consent for the genetic study (the assessment of chemosensitivity is part of the routine follow-up of these patients).

### Tidal breathing measurement and peripheral CO_2_ chemosensitivity assessment

Recordings of tidal breathing were performed as previously described [11]. Briefly, the participant was seated and breathed through a cushion-sealed face mask (Intersurgical, Wokingham, UK) fitted with elastic bands around the head and connected to a Validyne transducer (Northridge, CA). Therapy putty (Mobilis Rolyan, Petterson Medical) was used to minimize the dead space, preventing autostimulation of the chemoreflex by inspired CO_2_. The flow rate (pneumotachography), end-tidal PO_2_ (PETO_2_) and end-tidal PCO_2_ (PETCO_2_) were continuously monitored, and signals were digitized via the MP-100 system (Biopac System Inc., Santa Barbara, CA) at a rate of 50 Hz; these signals were stored for further analysis.

We used a constrained bivariate (minute ventilation V’E and PETCO_2_ of tidal ventilation recording) analytical model that allowed us to calculate the components of the controller and plant gains [11]. The model has previously been described in detail [12].

The model was fitted on the basis of the changes from baseline (mean) levels of the ventilatory parameters (V’E and PETCO_2_) that were obtained from tidal breathing measurements while awake; thus, the analyses were specifically related to chemosensitivity, and the steady-state controller gain (CG0) was assumed to assess peripheral CO_2_ chemosensitivity [11]. The two variables of interest were PETCO_2_ and CG0.

### Hyperoxic, hypercapnic ventilatory response testing

**Fig. 1:**
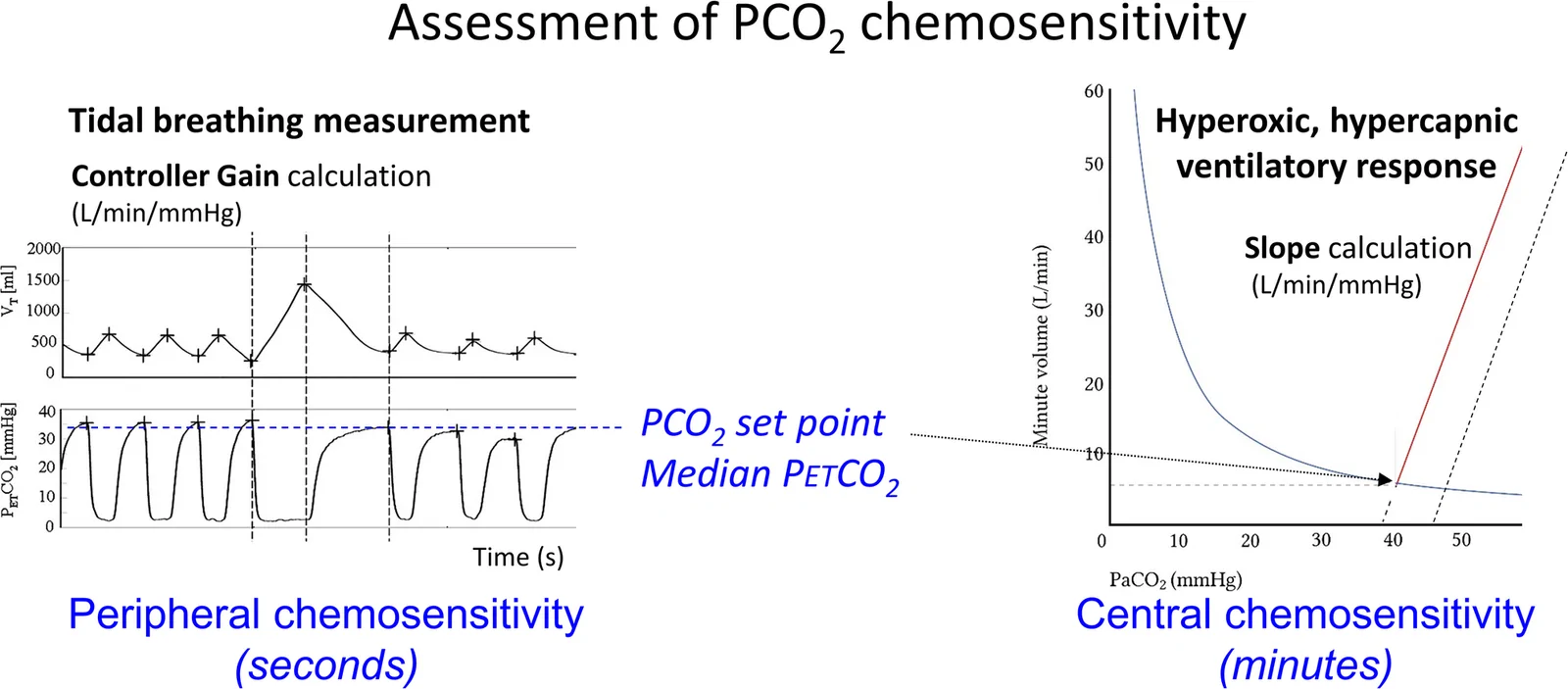
Assessment of CO_2_ chemosensitivity. The left panel shows the assessment of peripheral chemosensitivity while the right panel shows the assessment of central chemosensitivity. This figure has been adapted from reference [11]. The dashed line in the right panel summarizes the results of the L group, showing a rightward shift of resting PCO_2_ and ventilatory response threshold with no change in the response slope (central chemosensitivity)

After tidal breathing, the ventilatory response to hypercapnia was measured via a modification of the Read hyperoxic hypercapnic rebreathing technique, as previously described [11]. The participants rebreathed from a four-liter bag filled with a gas mixture with an initial composition of 95% O_2_ and 5% CO_2_. The ventilatory response to hypercapnia was expressed as V’E versus the end-tidal CO_2_:CO_2_ response slope. The hypercapnic ventilatory response by this rebreathing method under hyperoxia tests central ventilatory chemosensitivity since hyperoxia silences the response of peripheral chemoreceptors to CO_2_ [11]. At the end of the test, the maximal PETCO_2_ value was recorded as a quality criterion. Thus, the two variables of interest were the CO_2_ response slope and maximal PETCO_2_. The Fig. 1 describes these assessments of CO_2_ chemosensitivity.

### DNA collection and analysis: *5-HTT* polymorphism determination.

The DNA analysis was carried out by the Genetics Unit of our hospital. A blood sample was collected into ethylenediaminetetraacetic acid from each participant, and DNA was extracted from peripheral blood leukocytes according to standard protocols. The *5-HTT/SLC6A4* gene promoter region was amplified via polymerase chain reaction with the oligonucleotide primers described in 1996 by Heils and colleagues [7]: F_5’-GGCGTTGCCGCTCTGAATGC-3’ and R_5’-GAGGGACTGAGCTGG-ACAACCAC-3’. These primer sets amplify 484/528 fragments corresponding to short (s) and long (l) alleles, respectively. The PCR was carried out in a total volume of 25 µL consisting of 25 ng of genomic DNA, 20 pmol of each primer, 5 nmol of deoxynucleotide triphosphates, and 0.2 µL of FastStart Taq DNA Polymerase with GC-rich buffer (Roche Diagnostics GmbH). The annealing temperature was 68 °C for 30 s, the sample was extended at 72 °C for 1 min, and the sample was denatured at 95 °C for 40 s for 35 cycles. The amplification products were separated via automated electrophoresis on a D1000 ScreenTape TapeStation 4200 platform (Agilent, Santa Clara, CA, USA). The results were analyzed with proprietary software (TapeStation Analysis Software, Agilent).

### Sample size calculation and statistical analyses

For analyses, two groups were formed on the basis of the presence (S group: ss or sl) or absence (L group: ll) of the s variant of the serotonin transporter. Dichrophic grouping was used because serotonin uptake in human lymphoblastoid cells is similar for cells carrying either one or two copies of the short variant of the serotonin transporter [10]. Cells homozygous for the 5- serotonin transporter long variant (L group) have approximately 2-fold greater uptake [10]. We hypothesized that the L group would have a frank decrease of 0.30 L/min/mmHg in their CO_2_ response slope (mean value to null value) compared with the S group, with a standard deviation of 0.20 L/min/mmHg and a proportion of subjects in the L group of 0.25. The sample size needed with a Type I error rate of 0.05 and a Type II error rate of 0.20 was 19 (5 and 14: normal approximation using the Z statistic instead of the T statistic). We decided to include at least 20 subjects.

 Owing to the restricted sample size, the results are expressed as medians and percentiles, and two-group comparisons were made via the Mann‒Whitney test. A P value < 0.05 was considered significant. 

## Results

Twenty-three children (11 girls), median age 11 years [9; 16], with *PHOX2B* pathogenic variants were ultimately included: 7 with 20/25 PARM, 6 with 20/26 PARM, 6 with 20/27 PARM, 1 with 20/33 PARM and 3 with non-PARM. The distributions of *5-HTT* genotypes in the children with CCHS revealed that the percentages of the ll, ls, and ss genotypes were 5/23 (22%, 95% CI: 7–44), 14/23 (61%, 95% CI: 39–80) and 4/23 (17%, 95% CI: 5–39), respectively. The distributions of these genotypes were not significantly different among the three *PHOX2B* variant groups (moderate: 20/25/20/26 versus severe: 20/27; 20/33 versus non-PARM; *p* = 0.558).

**Fig. 2:**
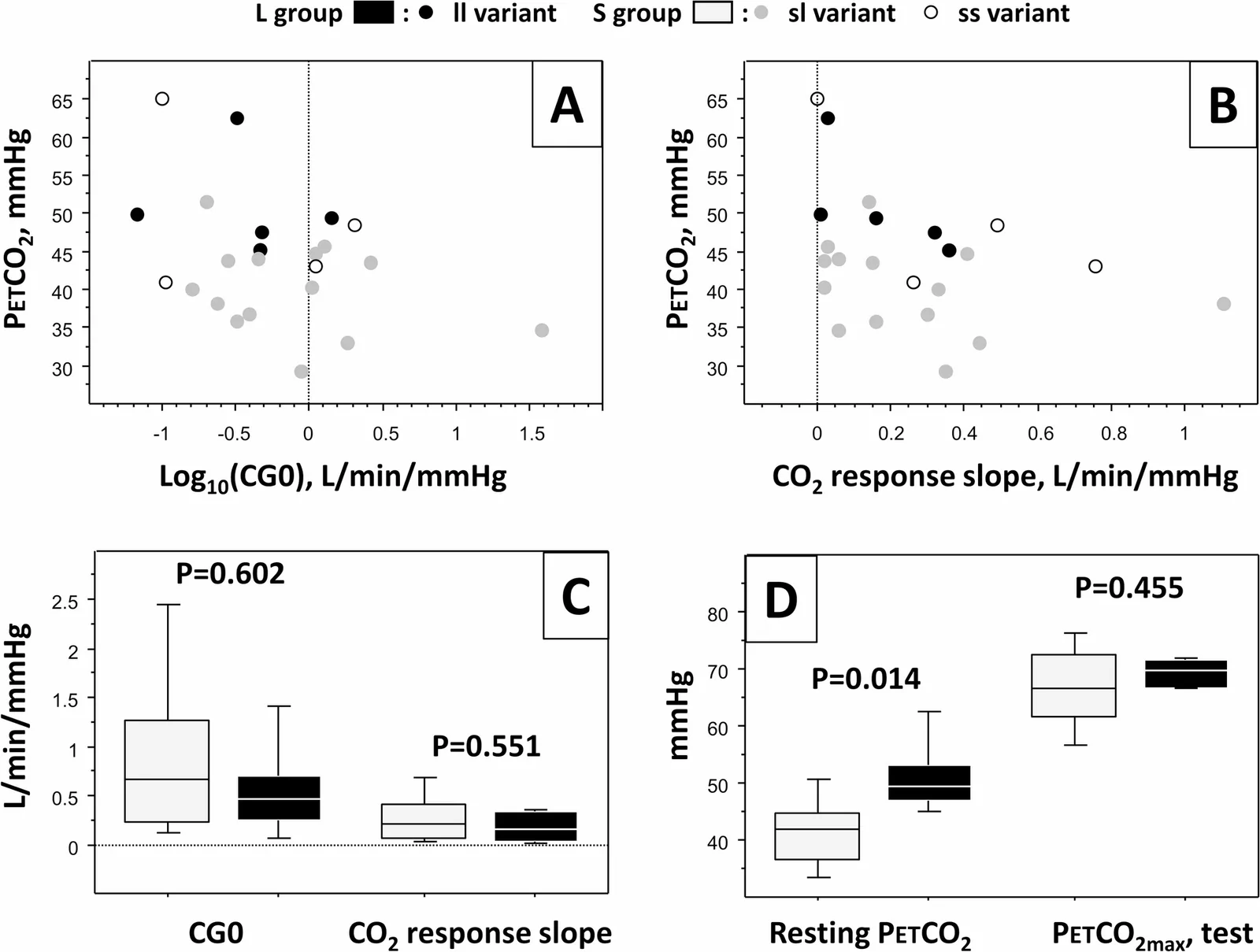
Results of ventilatory control assessments. Both peripheral (Log_10_CG0) and central (CO_2_ response slopes of the three *5-HTT* genotypes (black circles: ll, *n* = 5; gray circles: sl, *n* = 14; white circles: ss, *n* = 4) are provided in panels **A** and **B**, respectively, according to their resting PETCO_2 values_. Panels **C** and **D** describe chemosensitivity and resting PETCO_2_ according to the L group (black boxes; ll variant, *n* = 5) or S group (gray boxes, sl or ss variants, *n* = 18). Box and whisker plots show the median, 25th and 75th percentiles, and 10th and 90th percentiles

The results of their chemosensitivity and PETCO_2_ are provided in Fig. 2, which shows that both central (CO_2_ response slope) and peripheral (CG0) CO_2_ chemosensitivities were not significantly different when comparing the S group (*n* = 18) and the L group (*n* = 5), whereas their resting PETCO_2_ values were different; the L group presented a higher PETCO_2_ value than did the S group.

## Discussion

The main result of our study was that the CO_2_ chemosensitivity of children with CCHS was not related to *5-HTT* transporter polymorphisms, whereas these polymorphisms affected their resting PETCO_2_ values.

### CO_2_ chemosensitivity

The proportions of *5-HTT* polymorphisms were those expected in the French population. Figure 2 (panels A and B) shows that we reproduced our previous findings to some extent, showing that both peripheral and central chemosensitivity contributed to resting PETCO_2_ [11]. Clearly, no trend toward decreased chemosensitivity was observed in the L group, suggesting that the serotonin concentration in the raphe was not a main determinant of residual CO_2_ chemosensitivity in patients with CCHS.

### CO_2_ set point

Children with CCHS in the L group, who have decreased serotonin brain activity, had increased resting PETCO_2,_ suggesting that the raphe could contribute to the CO_2_ set point. In our previous study of the CCHS, 61% of the variance in PETCO_2_ was explained by peripheral and central chemosensitivities [11]. Thus, our present results may suggest that serotonin activity contributes to the remaining 39% of the variance. One may wonder whether the modified resting CO_2_ set point could be associated with normal CO_2_ chemosensitivity. In hyperventilation syndrome with sustained hypocapnia, Jack et al. reported that a decreased CO_2_ set point was associated with a normal hyperoxic hypercapnic response [13]. Hyperventilation syndrome is often associated with anxiety [14]. Carriers of the short allele of the 5*-HTT* gene have increased anxiety-related temperamental traits [10], suggesting a relationship between a decreased CO_2_ set point and the short allele of the *5-HTT* gene. Furthermore, the long-term administration of serotonin reuptake inhibitors in rats has been associated with hyperventilation [15], and very recently, Carr et al. reported that acute inhibition of serotonin reuptake in healthy subjects increased baseline ventilation and reduced the central chemoreceptor-mediated ventilatory response to CO_2_ [16]. Thus, by contrast, decreased serotonin brain activity would effectively be associated with hypoventilation, as evidenced in our study, whereas the effect on the central CO_2_ chemoreflex would be difficult to predict owing to discordances in the literature.

### Interpretation of the results and hypotheses

We hypothesize that some of the controversial results concerning CO_2_ chemosensitivity could be related to the method of its assessment. Breath-by-breath modifications of PETCO_2_ assess rapid peripheral CO_2_ chemosensitivity (seconds), whereas the hyperoxic hypercapnic response test assesses central CO_2_ chemosensitivity (minutes), which is likely related to the retrotrapezoid nucleus [17]. Serotonergic neurons could be involved in the chemosensitive regulation of longer kinetics (hours) and the resulting CO_2_ set point, such as during the night, when the serotonin effect is silenced, participating in nighttime hypoventilation. Similarly, in a goat model of carotid body denervation, analyses of brainstem tissues revealed a midline raphe-specific reduction in the percentage of tryptophan hydroxylase-expressing neurons and a reduction in serotonin transporter density in five medullary respiratory nuclei, suggesting that serotonin withdrawal is responsible for hypercapnia following carotid body denervation [18]. Therefore, it would be interesting to assess whether selective serotonin reuptake inhibitors are able to stimulate ventilation in patients with CCHS belonging to the ll variant genotype of *5-HTT*.

### Limitations of the study

It has to be stated that the effect of these polymorphisms on *5-HTT* expression in the human brainstem has not yet been assessed and that a decreased serotonin concentration in the raphe in the ll variant remains speculative. Furthermore, the small sample size of the study and the potential sensitivity to outliers, is another limitation.

## Conclusions

In conclusion, *5-HTT* gene polymorphisms do not seem to affect residual peripheral and central CO_2_ chemosensitivity in CCHS patients, at least when assessed over minutes, but may influence the PCO_2_ set point.

## Data Availability

The datasets generated and/or analysed during the current study are not publicly available due to restrictions related to our institution AP-HP, but are available from the corresponding author on reasonable request.

## Abbreviations

CCHS: Congenital central hypoventilation syndrome
PETCO2: End-tidal PCO2
PETO2: End-tidal PO2
V’E: Minute ventilation
PHOX2B: Paired-like homeobox 2B gene
PARM: Polyalanine repeat mutation
5-HT: Serotonin
5-HTT: Serotonin transporter
CG0: Steady-state controller gain
